# Exosomes derived from HCC cells induce sorafenib resistance in hepatocellular carcinoma both in vivo and in vitro

**DOI:** 10.1186/s13046-016-0430-z

**Published:** 2016-09-30

**Authors:** Zhen Qu, Junhua Wu, Junyi Wu, Dongjun Luo, Chunping Jiang, Yitao Ding

**Affiliations:** 1 Department of Hepatobiliary Surgery, Affiliated Drum Tower Hospital of Nanjing University Medical School, 210008 Nanjing, Jiangsu Province China; 2 Jiangsu Province’s Key Medical Center for Hepatobiliary Surgery, 210008 Nanjing, Jiangsu Province China; 3 Jiangsu Key Laboratory of Molecular Medicine, Medical School, Nanjing University, 210093 Nanjing, Jiangsu Province China; 4 Department of Hepatobiliary Surgery, Drum Tower Clinical College of Nanjing Medical University, 210008 Nanjing, Jiangsu Province China

**Keywords:** Exosomes, Sorafenib resistance, Hepatocellular carcinoma

## Abstract

**Background:**

Exosomes are carriers of intercellular information and regulate the tumor microenvironment. They play an important role in drug resistance by transporting RNA molecules and proteins. However, their effects on sorafenib resistance in hepatocellular carcinoma (HCC) are not completely understood.

**Methods:**

Exosomes were isolated from two invasive hepatoma cell lines (MHCC-97 L and MHCC-97H), and their roles in regulating sorafenib resistance in liver cancer cells as well as the underlying molecular mechanisms were determined. The exosomes were analyzed by TEM (transmission electron microscopy), DLS (dynamic light scattering) and Western blotting. Cell viability, cell death and the effects of exosomes on the HGF/c-Met/Akt signaling pathway in cancer cells were analyzed by MTT assays, FACS analysis and Western blotting, respectively. Moreover, the effects of exosomes on sorafenib resistance in vivo were investigated using a subcutaneous transplantation tumor model in athymic nude mice.

**Results:**

Exosomes derived from HCC cells were of the expected size and expressed the exosomal markers CD9 and CD63. They induced sorafenib resistance in vitro by activating the HGF/c-Met/Akt signaling pathway and inhibiting sorafenib-induced apoptosis. They also induced sorafenib resistance in vivo by inhibiting sorafenib-induced apoptosis. Moreover, exosomes derived from highly invasive tumor cells had greater efficacy than that of exosomes derived from less invasive cells.

**Conclusions:**

These data reveal the important role of HCC cell-derived exosomes in the drug resistance of liver cancer cells and demonstrate the intrinsic interaction between exosomes and their targeted tumor cells. This study suggests a new strategy for improving the effectiveness of sorafenib in treating HCC.

## Background

Hepatocellular carcinoma (HCC) is the fifth most common cancer and second leading cause of cancer-related deaths worldwide, resulting in 700,000 deaths annually [1]. Currently, surgical resection is the major treatment modality for early-stage liver cancer [2]; however, most patients are diagnosed in the advanced stage when treatments have little effect. Currently, the 5-year survival rate of patients with HCC is less than 20 % [3].

Phase III clinical trials showed that sorafenib, a multi-target tyrosine kinase inhibitor that decreases tumor cell proliferation and angiogenesis [4, 5], improved the overall survival of patients with advanced HCC [6]. Therefore, in 2007, it was approved by the US Food and Drug Administration as a molecular targeted drug for unresectable liver cancer. Although sorafenib is currently the only medication approved for the treatment of liver cancer, its therapeutic effects are affected by several signaling pathways, such as the reactivation of ERK signaling and inhibition of MAPK [7, 8]. Previous studies have suggested that gastrointestinal stromal tumor cell-derived exosomes contain oncogenic KIT, and their transfer and uptake by the surrounding smooth muscle cells led to enhanced AKT and MAPK signaling and enhanced tumor cell invasion [9].

Exosomes play important roles in the exchange of biological information as substance transport carriers and in regulation of the cellular microenvironment by delivering a variety of biological molecules, including mRNAs, miRNAs, and proteins [10–13]. Tumor cell-derived exosomes are involved in the regulation of the epithelial-mesenchymal transition, tumor angiogenesis, tumor metastasis, and radioresistance [14–17]. Docetaxel and cisplatin promote the secretion of exosomes from tumor cells; the exosomes alter drug sensitivity by releasing molecules such as mRNAs and miRNAs into neighboring cells [18, 19]. Safei et al. [20] found that cisplatin-resistant ovarian cancer cells discharged anticancer drugs through exosomes and expressed higher levels of the transporter proteins MRP2, ATP7A and ATP7B than those of cisplatin-sensitive cells, suggesting that exosomes play a key role in resistance to chemotherapy. However, whether HCC cell-derived exosomes are involved in sorafenib resistance in liver cancer cells and the potential underlying mechanisms are currently unclear.

In this study, we investigated whether HCC cell-derived exosomes mediate sorafenib resistance in HCC cells and determined the potential molecular mechanisms underlying this process. We found that HCC cell-derived exosomes enhanced sorafenib resistance in liver cancer in vitro by inhibiting sorafenib-induced apoptosis via activation of the HGF/c-Met/Akt signaling pathways. These findings reveal the important role of HCC cell-derived exosomes in the drug resistance of liver cancer cells and demonstrate the intrinsic interaction between exosomes and their targeted tumor cells. The results of this study may provide a new strategy for improving the effectiveness of sorafenib in treating liver cancer.

## Methods

### Cell lines and cell culture

The human HCC cell line SMMC-7721 was purchased from the Cell Bank of the Chinese Academy of Sciences (Shanghai, China), and the MHCC-97H, MHCC-97 L and LO2 cell lines were obtained from the Liver Cancer Institute, Fudan University (Shanghai, China). SMMC-7721, MHCC-97H and MHCC-97 L cells were cultured in complete DMEM containing 10 % fetal bovine serum (FBS) and penicillin (100 U/mL). LO2 cells were cultured in RPMI-1640 medium containing 10 % FBS, penicillin (100 U/mL), and streptomycin (100 μg/mL). All cells were incubated at 37 °C in humidified air with 5 % CO_2_.

### Extraction of exosomes

MHCC-97H, MHCC-97 L and LO2 cell lines were cultured in media with 10 % exosome-free FBS (by ultracentrifugation overnight). After 48 h, cell culture media were collected, and exosomes were isolated from the supernatant by differential centrifugation as previously described [21]. Finally, the protein content of the concentrated exosomes was determined using a BCA protein assay kit (Thermo Scientific, USA). CD9, CD63 and GAPDH (antibodies for CD9, CD63 and GAPDH were obtained from Cell Signaling Technology, Beverly, MA, USA) expressions were measured using Western blot analyses. The aliquots were stored at -80 °C.

### Transmission electron microscopy (TEM) and size distribution analysis

The extracted pellets were observed by TEM as previously described [22]. Approximately 10 μL of purified exosomes was fixed in 1 % glutaraldehyde for 10 min, washed, and contrasted in 2 % uranyl acetate. Images were obtained by TEM (JEM-2100, Jeol, Japan). Scanning ion occlusion sensing analysis was performed using a Zetasizer Nano ZS90 instrument (Malvern, UK) according to the manufacturer’s instructions. Isolated exosome samples were resuspended in PBS. All samples were measured with parameters of 44.5 mm and 0.64 V voltage using NP100 membranes. Samples were calibrated by CPC100 standard particles diluted 1000-fold under identical settings.

### Animal model

All animal procedures were performed according to national guidelines and approved by the Animal Care Ethics Committee of Nanjing Drum Tower Hospital. Twenty-five male BALB/c nu/nu mice (4–6 weeks old) were purchased from the Laboratory Animal Center of Shanghai, Academy of Science. First, all mice received subcutaneous injections of SMMC-7721 cells in the right armpit after infiltration anesthesia with lidocaine (0.25 %, Nanjing Drum Tower Hospital) (1 × 10^7^ cells in 200 μL PBS per mouse). When the tumors reached a volume of 50–100 mm^3^ (15 days after subcutaneous injections of tumor cells), twenty-five mice were randomly divided into five groups (*n* = 5, the control group, sorafenib group, sorafenib + LO2-exosome group, sorafenib + MHCC-97 L-exosome group, and sorafenib + MHCC-97H-exosome group). The sorafenib group was intraperitoneally injected with sorafenib (100 mg/kg, Selleck, USA) daily for 10 days. The sorafenib + LO2-exosome group, sorafenib + MHCC-97 L-exosome group, and sorafenib + MHCC-97H-exosome group were intraperitoneally injected with sorafenib (100 mg/kg, Selleck, USA) daily and subcutaneously injected with LO2, MHCC-97 L and MHCC-97H cell-derived exosomes (100 μg total protein in 100 μL volume, in the vicinity of the subcutaneous tumors), respectively, every day for 10 days. The control group was intraperitoneally injected with 0.4 % dimethyl sulfoxide (DMSO) in PBS (the vehicle for sorafenib) and subcutaneously injected with PBS (the vehicle for exosome) daily for 10 days. The mice were examined every 2 days, and all mice sacrificed by cervical dislocation under general anesthesia with chloral hydrate (5 %, 100 μL/10 g) 25 days after the subcutaneous injections of tumor cells.

### Cell viability analysis

Cell viability was monitored using MTT assays. Briefly, 5 × 10^3^ cells were cultured on 96-well plates. After incubation with sorafenib alone or sorafenib and exosomes for 24 or 48 h, 20 μL MTT solution (0.5 %) was added to the medium and incubated for 4 h. Then, the medium was removed, and 150 μL DMSO was added to each well to dissolve the insoluble formazan product. The absorbance of the colored solution was measured at 490 nm with a spectrophotometer. All of the experiments were performed in triplicate.

### Western blot analysis

HCC cells were lysed with RIPA peptide lysis buffer (Beyotime Biotechnology, China) containing 1 % protease inhibitors (Thermo Scientific, USA). Equal amounts of proteins were loaded and resolved using 10 % sodium dodecyl sulfate-polyacrylamide gel electrophoresis (SDS-PAGE). Antibodies for CD9, CD63, GAPDH, caspase-9, caspase-3, PARP, c-Met, p-Met, AKT, p-Akt, VEGFR2 and p-VEGFR2 were obtained from Cell Signaling Technology (Beverly, MA, USA). The Met inhibitor crizotinib (PF-02341066) and the p-Akt inhibitor MK-2206 2HCl were purchased from Selleck (Selleck Chemicals, China). After incubation with horseradish peroxidase-conjugated secondary antibodies, protein bands were visualized using enhanced chemiluminescence (Millipore, USA).

### Fluorescence microscopy analysis of exosome internalization

MHCC-97H-derived exosomes were labeled with CM-DIL (Sigma-Aldrich, St. Louis, MO, USA) as follows. Two microliters of CM-DIL was added to 100 μg of MHCC-97H-derived exosomes in a total of 1 mL of diluent and incubated for 15 min at room temperature, and the mixture was added to 18 mL of PBS and centrifuged at 120,000 g for 2 h at 4 °C. The supernatant was removed, and the pellet was resuspended in 20 mL of PBS and centrifuged at 120,000 g for 2 h at 4 °C. The pellet containing CM-DIL-labeled exosomes was resuspended in 200 μL of PBS medium. SMMC-7721-GFP cells were cultured in a four-chamber slide to 80 % confluency. The medium was added with PBS medium containing CM-DIL-labeled exosomes, and cells were incubated for 4 h at 37 °C in 5 % CO_2_. After incubation, the cells were washed twice with PBS and fixed in polyformaldehyde for 10 min. The slide was mounted with ProLong Gold Antifade Reagents, and internalization of the exosomes was analyzed using fluorescence microscopy.

### TUNEL assay

Subcutaneous tumor samples from nude mice were paraffin-sectioned by routine methods. Apoptotic cells were visualized using terminal deoxynucleotidyl transferase-mediated dUTP nick end labeling (TUNEL) assays. The TUNEL procedure was performed using an in situ cell death detection kit (Keygentec, KGA7025, China) according to the manufacturer’s instructions. Briefly, the fixed cells were incubated in 20 mg/mL proteinase K for 30 min at room temperature, followed by incubation with equilibration buffer for 10 s. The cells were washed with PBS and incubated with streptavidin-FITC at 37 °C for 1 h in a humidified chamber. Then, the cells were washed with PBS again and incubated with anti-digoxigenin conjugate (rhodamine antibody) and counterstained with DAPI. The microscopic images of the cells were visualized by fluorescence microscopy (BX41, Olympus, Japan).

### Enzyme-linked immunosorbent assay (ELISA)

The concentration of hepatocyte growth factor (HGF) in the cellular supernatant was detected using a human HGF ELISA kit (ExCell, Shanghai, China) following the manufacturer’s instructions. Briefly, after the cells were treated with sorafenib and HCC cell-derived exosomes for 48 h, the media was collected and centrifuged at 5000 rpm for 5 min. Total media with 10 % FBS was used as the control.

### Statistical analysis

All data are expressed as the mean ± SD from three individual experiments. Differences between groups were determined using Student’s *t-*test or analysis of variance (ANOVA). *P* values less than 0.05 were considered statistically significant.

## Results

### Extraction and characterization of HCC cell-derived exosomes

**Fig. 1:**
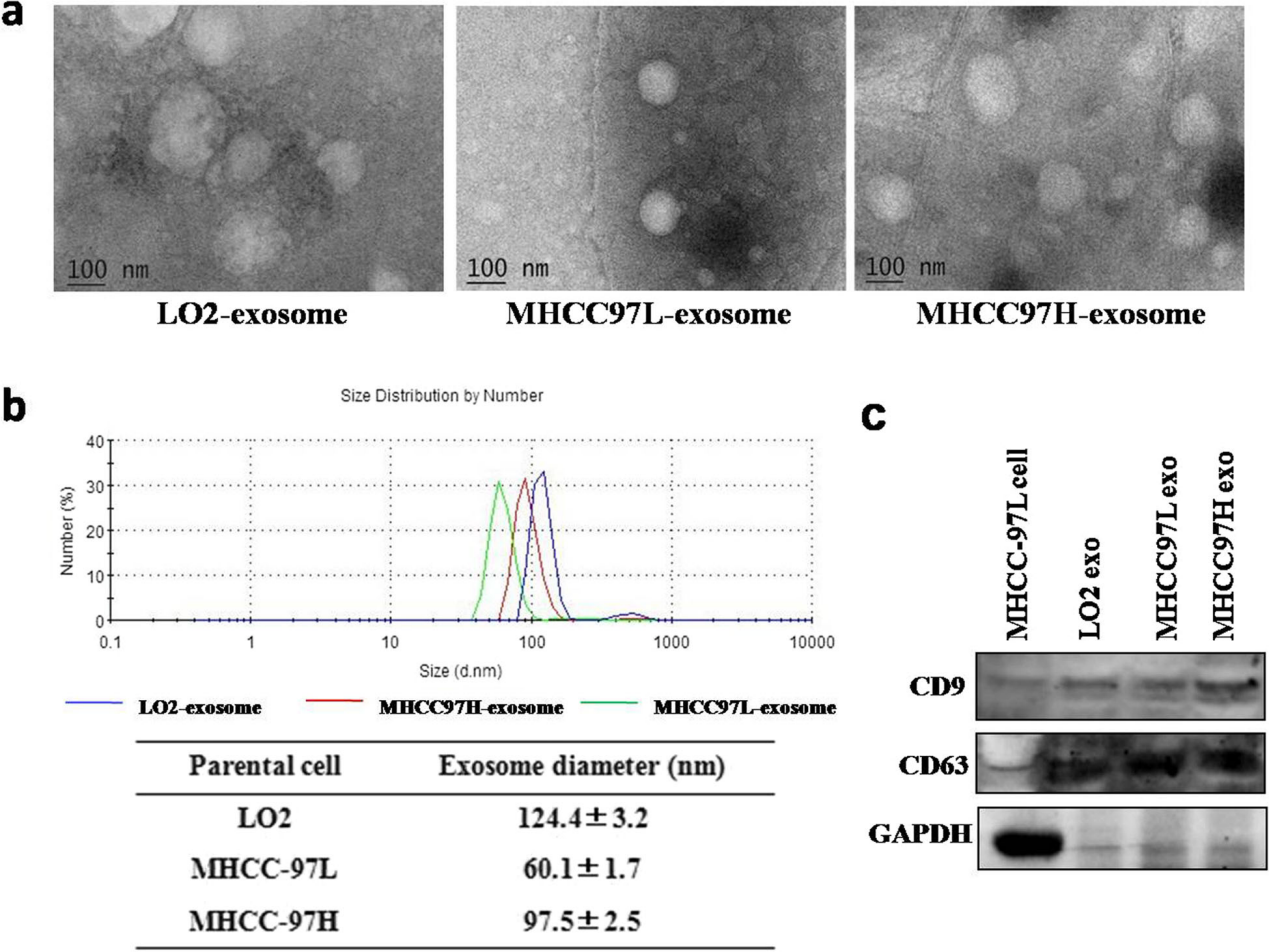
Characterization of exosomes derived from different cell lines. **a** TEM confirmed that the final pellets from ultracentrifugation were exosomes (scale bar, 100 nm). **b** Size distribution analysis of purified exosomes by DLS (Nano-ZS90, Malvern). **c** Exosomal markers (CD9, CD63) were analyzed using Western blotting and are present in cells and exosomes (GAPDH was used as an internal reference)

To determine the effects of exosomes from different sources on sorafenib resistance in HCC cells, we first used ultracentrifugation to isolate exosomes from the supernatants of two hepatoma cell lines (MHCC-97H and MHCC-97 L) with different invasive potential and a non-invasive immortalized liver cell line (LO2). MHCC-97H has a higher invasive potential than that of MHCC-97H, and LO2 is a normal non-invasive liver cell line [23]. The exosomes were round in shape with diameters of 40–150 nm, as determined by TEM and DLS (Nano-ZS90, Malvern) (Fig. 1a, b), and expressed the exosomal markers CD9 and CD63 (Fig. 1c).

### HCC cell-derived exosomes can be taken up and internalized by hepatoma cells

**Fig. 2:**
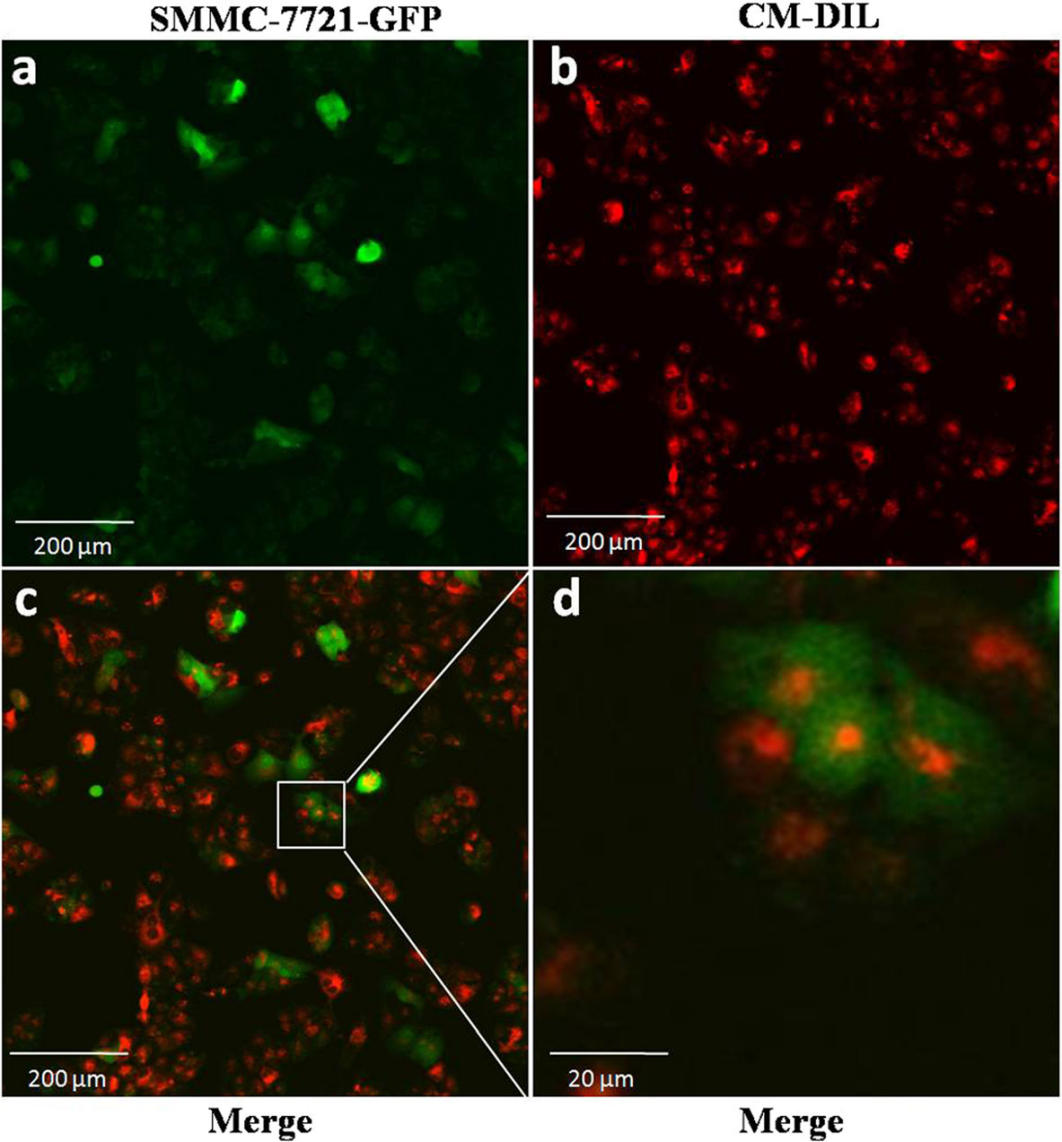
Internalization of MHCC-97H-derived exosomes in SMMC-7721-GFP cells. SMMC-7721-GFP cells in culture were incubated with MHCC-97H-derived exosomes labeled with CM-DIL (red). Cells were fixed with polyformaldehyde and mounted with ProLong Gold Antifade Reagent, as described in Materials and Methods. Low-magnification images of SMMC-7721-GFP cells incubated with exosomes (**a**, **b**, **c**). High-magnification images of SMMC-7721-GFP cells incubated with exosomes (**d**). MHCC-97H-derived exosomes were shown to be internalized in the cytoplasm of SMMC-7721-GFP cells

To examine the potential uptake and internalization of exosomes by SMMC-7721 cells, we labeled exosomes derived from MHCC-97H cells with a fluorescent dye, CM-DIL, as described in Materials and Methods. CM-DIL-labeled exosomes were incubated with SMMC-7721-GFP cells for 4 h, and localization of the exosomes was assessed by fluorescence microscopy (Fig. 2). CM-DIL-labeled exosomes were internalized as endosome-like vesicles in the cytoplasm of SMMC-7721-GFP cells (Fig. 2c, d). These studies indicate that HCC cell-derived exosomes can be taken up and internalized by HCC cells.

### HCC cell-derived exosomes induce sorafenib resistance in hepatoma cells in vivo

**Fig. 3:**
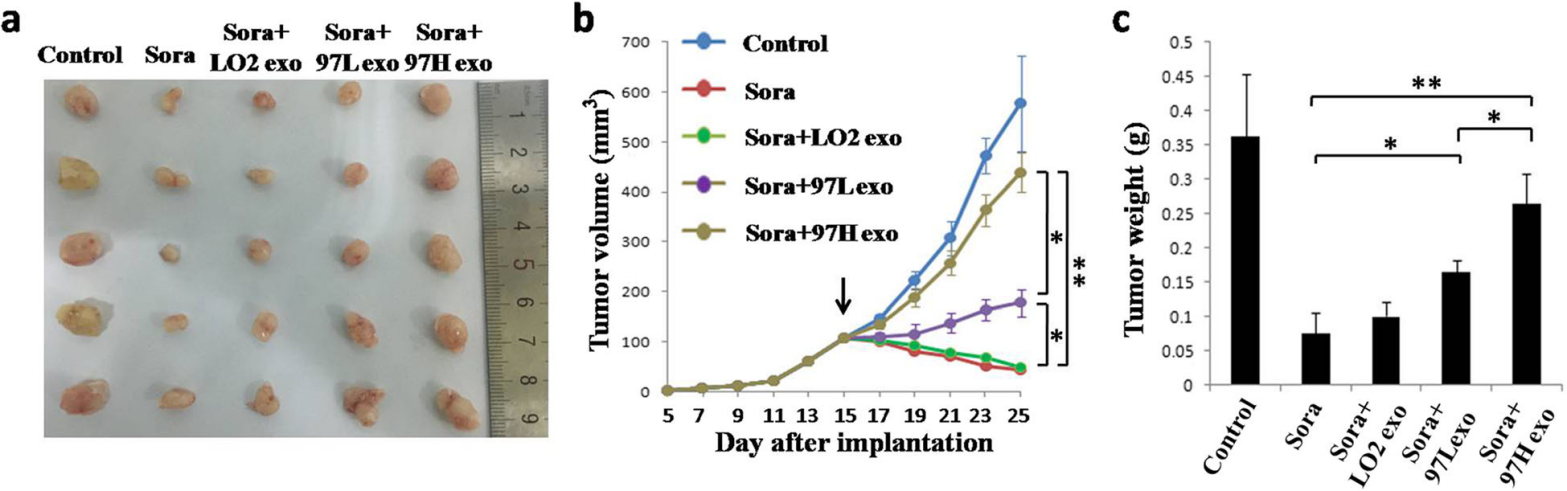
HCC cell-derived exosomes induce resistance to sorafenib in hepatoma carcinoma cells in vivo. **a** Tumors from mice treated with PBS (Control), sorafenib (Sora), sorafenib + LO2-exosomes (Sora + LO2 exo), sorafenib + MHCC-97 L-exosomes (Sora + 97 L exo), and sorafenib + MHCC-97H-exosomes (Sora + 97H exo) at the end of the experiment. **b** Tumor growth curves in mice treated with PBS (Control), sorafenib (Sora), sorafenib + LO2-exosomes (Sora + LO2 exo), sorafenib + MHCC-97 L-exosomes (Sora + 97 L exo), and sorafenib + MHCC-97H-exosomes (Sora + 97H exo) (*n* = 5, * *P* <0.05, ** *P* <0.01). Treatment was initiated when tumors reached a volume of 50–100 mm^3^ (15 days after subcutaneous injections of tumor cells). **c** The mean weight of the tumors from mice treated with PBS (Control), sorafenib (Sora), sorafenib + LO2-exosomes (Sora + LO2 exo), sorafenib + MHCC-97 L-exosomes (Sora + 97 L exo), and sorafenib + MHCC-97H-exosomes (Sora + 97H exo) at the end of the experiment (*n* = 5, * *P* <0.05, ** *P* <0.01)

To determine whether HCC cell-derived exosomes can induce sorafenib resistance in liver cancer in vivo, we established a subcutaneous xenograft model in nude mice and injected sorafenib together with LO2-, MHCC-97 L-, or MHCC-97H-derived exosomes into the mice. As shown in Fig. 3a, the tumors in mice treated with sorafenib plus MHCC-97 L- or MHCC-97H-derived exosomes were significantly larger than those in mice treated with sorafenib alone or sorafenib plus LO2-derived exosomes, indicating that invasive HCC cell-derived exosomes inhibit the therapeutic effects of sorafenib and promote tumor growth. Figure 3b-c shows the tumor volume and weight of each group. The tumor volume and weight of mice treated with sorafenib plus exosomes derived from MHCC-97H cells were approximately 5-fold greater than those in mice treated with sorafenib alone (Fig. 3b, c). Fig. 3c also demonstrates that tumors in mice treated with sorafenib plus MHCC-97H-derived exosomes were significantly larger than those in mice treated with sorafenib plus MHCC-97 L-derived exosomes, indicating that exosomes derived from a more invasive HCC cell line showed greater inhibition of the chemotherapeutic effects of sorafenib and stronger promotion of tumor growth that those of exosomes derive from less invasive cell lines. Tumor volume or tumor weight in mice treated with sorafenib alone showed no significant difference from that in mice treated with sorafenib plus LO2-derived exosomes.

### HCC cell-derived exosomes induce sorafenib resistance of hepatoma cells in vitro

**Fig. 4:**
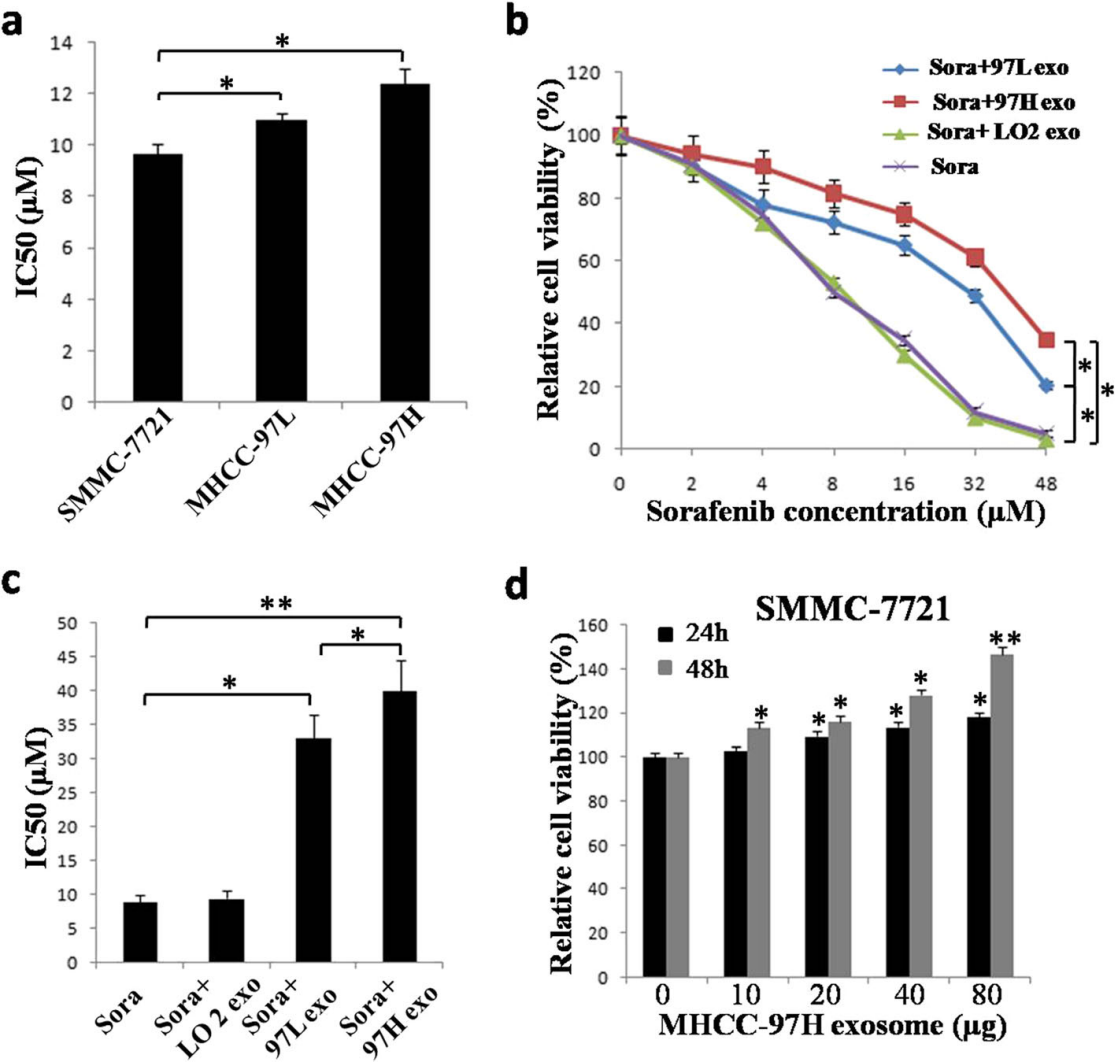
HCC cell-derived exosomes induced sorafenib resistance in SMMC-7721 cells. **a** IC_50_ values of sorafenib in SMMC-7721, MHCC-97 L and MHCC-97H cells determined by MTT assay (* *P* <0.05). The cells were treated with sorafenib for 48 h, and the sensitivity of SMMC-7721 cells to sorafenib was higher than that of MHCC-97 L and MHCC-97H cells (* *P* <0.05). **b** Cell viability was assessed by MTT assays. HCC cell-derived exosomes attenuated sorafenib-induced cell suppression. SMMC-7721 cells were treated with sorafenib at different concentrations with or without exosomes for 48 h (* *P* <0.05). **c** IC_50_ values for sorafenib in SMMC-7721 cells with or without exosomes determined by MTT assays. The SMMC-7721 cells were treated with sorafenib at different concentrations with or without exosomes for 48 h. The IC_50_ of sorafenib in SMMC-7721 cells in the HCC-derived exosome groups was notably elevated compared to that of the sorafenib alone group (* *P* <0.05; ** *P* <0.01). **d** MHCC-97H derived exosomes prevented the reduction of SMMC-7721 viability induced by sorafenib in a time- and dose-dependent manner (* *P* <0.05). Cell viability was assessed by MTT assays. The SMMC-7721 cells were treated with sorafenib at 10 μM with or without exosomes at different concentrations for 24 or 48 h

We used MTT assays to determine the half maximal inhibitory concentration (IC_50_) of sorafenib in different hepatoma cell lines. The results showed SMMC-7721 cells were more sensitive to sorafenib than MHCC-97 L and MHCC-97H cells, and MHCC-97H cells were the least sensitive to sorafenib among the three cell lines (Fig. 4a).

To determine if HCC cell-derived exosomes could induce sorafenib resistance in the SMMC-7721 HCC cell line in vitro, we used MTT assays to first confirm that sorafenib dose-dependently inhibited cell viability (Fig. 4b). Then, we cultured the cells in medium supplemented with sorafenib plus exosomes from different sources and examined cell viability again. We found that the addition of MHCC-97H- or MHCC-97 L-derived exosomes significantly inhibited the sorafenib-induced reduction of cell viability, but addition of LO2-derived exosomes did not have this effect, indicating that invasive HCC cell-derived exosomes inhibit the therapeutic effects of sorafenib and promote tumor cell growth. Furthermore, this effect was more dramatic with MHCC-97H- compared to that of MHCC-97 L-derived exosomes (*P* <0.05, Fig. 4b), indicating that more invasive HCC cell-derived exosomes show stronger promotion of HCC cell proliferation and inhibition of the chemotherapeutic effects of sorafenib. Moreover, MTT assays showed that the IC_50_ of sorafenib in SMMC-7721 cells treated with MHCC-97H- or MHCC-97 L-derived exosomes was notably elevated compared to that in the sorafenib alone group or the LO2-exosome group, and the IC_50_ of sorafenib in SMMC-7721 cells treated with MHCC-97H-derived exosomes was significantly higher than that in the MHCC-97 L-derived exosome group (*P* <0.05, Fig. 4c). Moreover, MHCC-97H-derived exosomes prevented the sorafenib-induced reduction of cell viability in a time- and dose-dependent manner (Fig. 4d).

### HCC cell-derived exosomes inhibit sorafenib-induced apoptosis

**Fig. 5:**
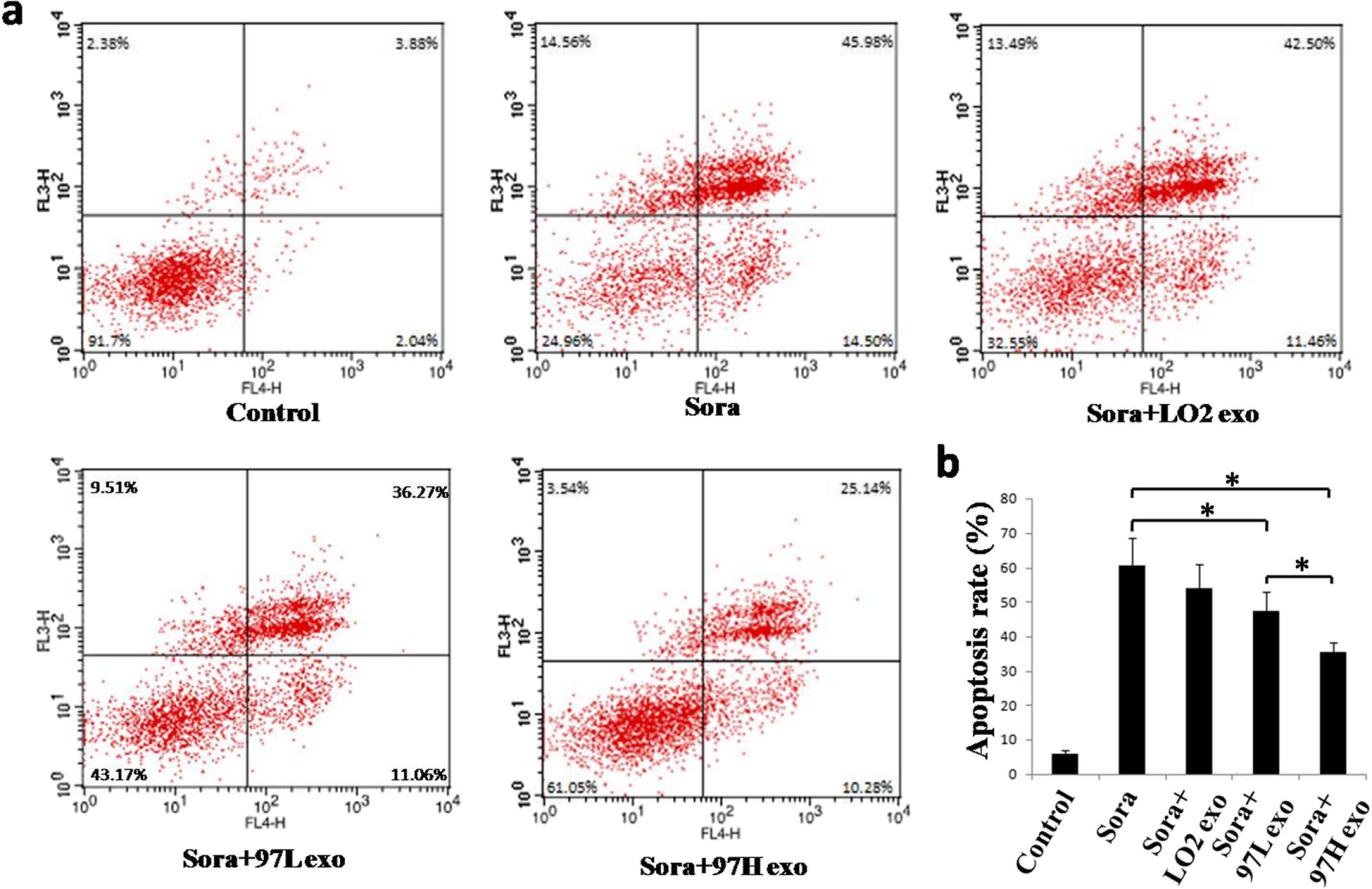
HCC cell-derived exosomes reverse sorafenib-induced apoptosis in hepatoma carcinoma cells in vitro. **a** Flow cytometric analyses of apoptotic cells ex vivo. The SMMC-7721 cells were exposed to sorafenib alone or sorafenib and exosomes for 48 h, collected and subjected to Annexin V/PI double staining, followed by FACS analyses. For each assay, 10,000 cells were analyzed. **b** The quantitative data are presented as the mean ± SD of triplicate experiments (* *P* <0.05)

To determine whether HCC cell-derived exosomes can affect sorafenib-induced cell apoptosis, we assessed sorafenib-induced apoptosis in hepatoma cells in the presence or absence of HCC cell-derived exosomes. SMMC-7721 cells were exposed to sorafenib for 48 h, and the percentage of apoptotic cells was analyzed using Annexin V-FITC/PI apoptosis staining. The apoptotic rate of SMMC-7721 cells in the sorafenib plus MHCC-97H- or MHCC-97 L-derived exosome groups was 35.42 % ± 2.82 % and 47.33 % ± 7.05 %, respectively, which was significantly lower than that in the sorafenib alone (60.48 % ± 9.42 %) or sorafenib plus LO2-derived exosome groups (53.96 % ± 8.64 %) (Fig. 5a, b).

**Fig. 6:**
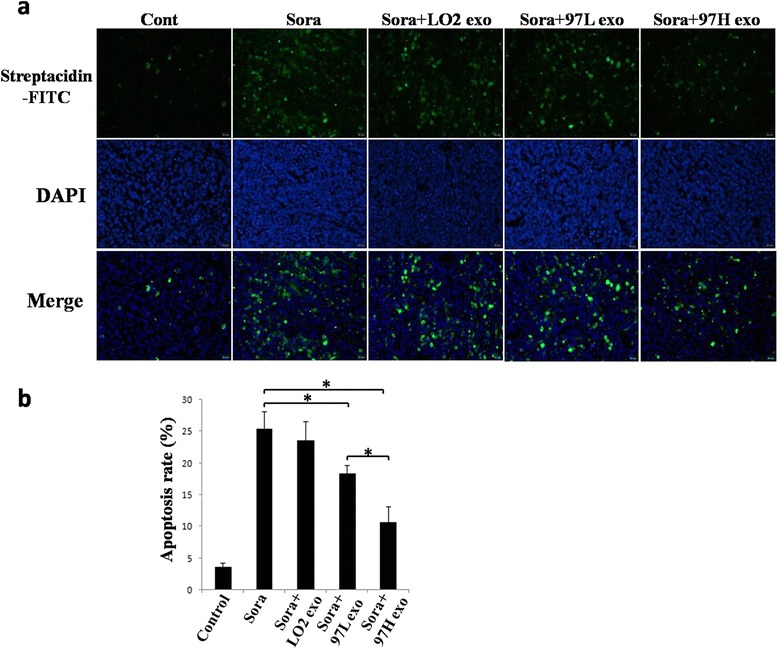
HCC cell-derived exosomes reverse sorafenib-induced apoptosis in hepatoma carcinoma cells in vivo. **a** Tumors from mice treated with PBS (Control), sorafenib (Sora), sorafenib + LO2-exosomes (Sora + LO2 exo), sorafenib + MHCC-97 L-exosomes (Sora + 97 L exo), and sorafenib + MHCC-97H-exosomes (Sora + 97H exo) were paraffin-embedded and sectioned, followed by staining of apoptotic cell by using TUNEL assays. **b** The number of TUNEL-positive cells notably decreased in the sorafenib + MHCC-97 L-exosome or sorafenib + MHCC-97H exosome groups compared to the those of the sorafenib alone or sorafenib + LO2-exosome groups (* *P* <0.05)

Moreover, to further elucidate the effect of HCC cell-derived exosomes on apoptosis in vivo, we used TUNEL staining. The results demonstrated that the number of apoptotic cells in the subcutaneous tumor tissues significantly increased after tumors were treated with sorafenib alone or sorafenib plus LO2-derived exosomes (25.41 ± 2.71, 23.61 ± 3.03 versus 3.65 ± 0.59, Fig. 6a, b). However, co-treatment with HCC cell-derived exosomes significantly reduced the apoptotic rate (10.64 % ± 2.44 %, 18.38 % ± 1.28 % versus 25.41 % ± 2.71 %, Fig. 6a, b). Furthermore, compared to the apoptotic rate in the sorafenib plus MHCC-97 L-derived exosome group, the apoptotic rate in the sorafenib plus MHCC-97H-derived exosome group was significantly higher (Figs. 5b and 6b).

**Fig. 7:**
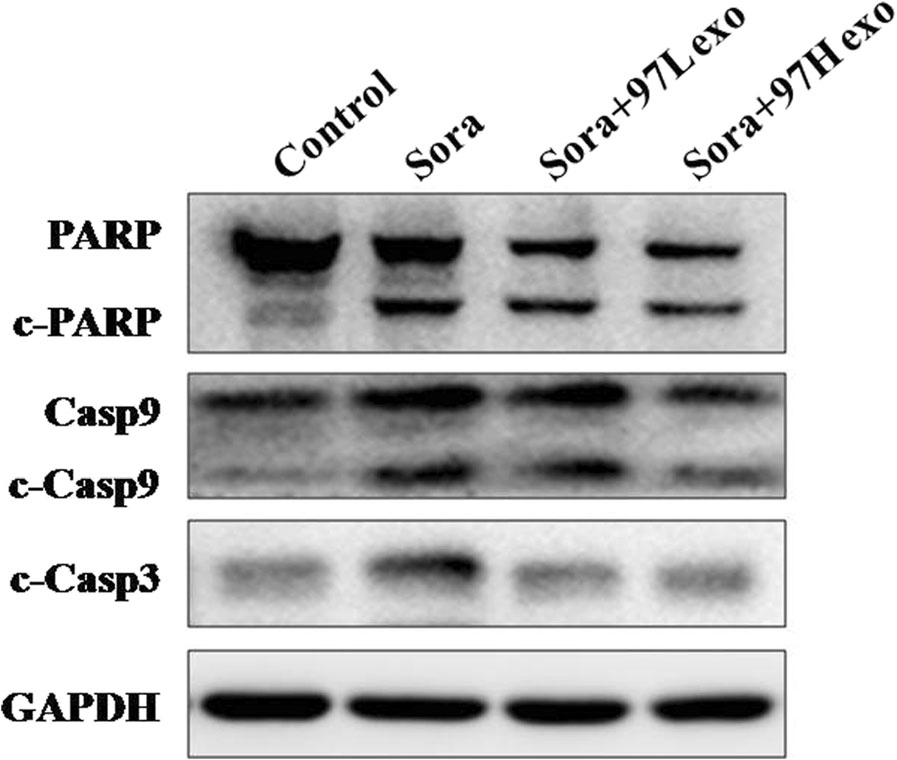
HCC cell-derived exosomes decreased cleavage of caspases and PARP. MHCC-97H-derived exosomes were more effective than MHCC-97 L-exosomes. Cell lysates were subjected to Western blotting after sorafenib alone or sorafenib and exosome administration for 48 h

Then, we assessed the effects of sorafenib alone or sorafenib plus exosomes from different sources on the levels of apoptotic proteins, such as cleaved caspase-9, caspase-3, and PARP, in SMMC-7721 cells. Treatment with sorafenib plus MHCC-97H- or MHCC-97 L-derived exosomes significantly reduced the levels of cleaved caspase-9, caspase-3, and PARP compared with that in the sorafenib alone group or sorafenib plus LO2-exosome group (Fig. 7), indicating that HCC cell-derived exosomes can partially reverse sorafenib-induced apoptosis. Interestingly, the effects of MHCC-97H-derived exosomes were more dramatic than those of MHCC-97 L-derived exosomes. Taken together, these results suggested that HCC cell-derived exosomes could reverse the induction of apoptosis by sorafenib in HCC, and more invasive HCC cell-derived exosomes showed a greater ability to reverse sorafenib-induced apoptosis.

### HCC cell-derived exosomes affect sorafenib resistance via the HGF/c-Met/Akt pathway

**Fig. 8:**
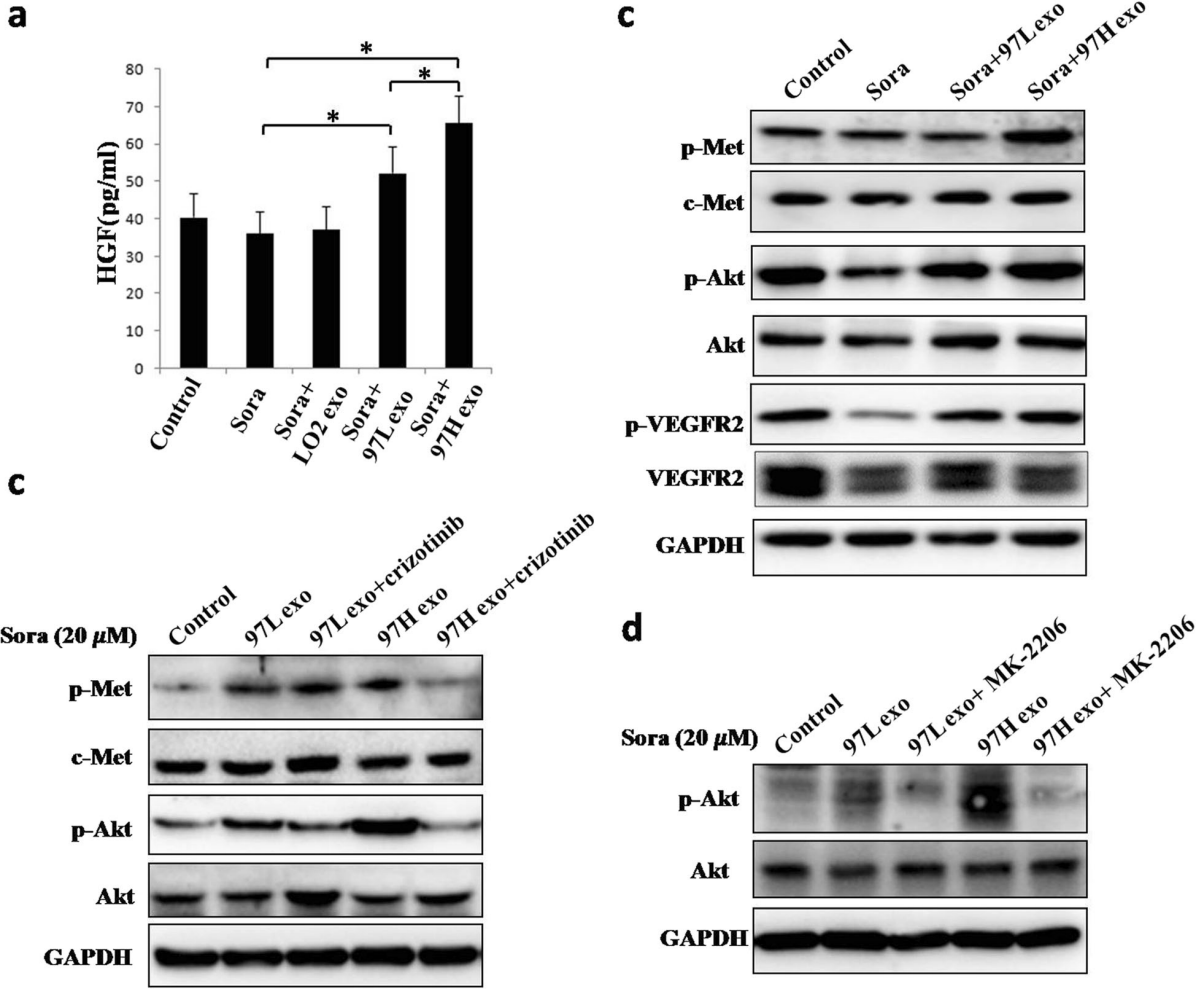
HCC cell-derived exosomes affect sorafenib resistance via the HGF/c-Met/Akt pathway. **a** Elevated HGF levels in the cell culture supernatant contributed to sorafenib resistance induced by HCC cell-derived exosomes. HGF concentrations in the supernatant were determined by ELISA. Treatment of cells with HCC cell-derived exosomes for 48 h significantly increased HGF levels in the cell culture supernatant compared to that of the sorafenib group. The effects of MHCC-97H-derived exosomes were greater than those of MHCC-97 L-derived exosomes (* *P* <0.05). **b** HCC cell-derived exosomes increased the level of phosphorylated Met, Akt and VEGFR2 compared with that of sorafenib alone. **c** The increase in phosphorylated c-Met and Akt induced by MHCC-97H and MHCC-97 L derived exosomes was reduced by the c-Met inhibitor crizotinib (sora: sorafenib). **d** The increase in the levels of phosphorylated Akt induced by MHCC-97H- and MHCC-97 L-derived exosomes was weakened by the p-Akt inhibitor MK-2206 (sora: sorafenib)

To explore the possible mechanisms by which HCC cell-derived exosomes induce sorafenib resistance, we also examined HGF levels in the supernatants of cells from different treatment groups using ELISAs. Previous reports have shown that exosomes can induce erlotinib resistance in breast cancer. Our results indicated that treatment with HCC cell-derived exosomes for 48 h increased HGF levels in the cell culture supernatant compared to those of the control group, and MHCC-97H-derived exosomes caused the most significant effects (*P* <0.05, Fig. 8a). Western blot analysis showed that treatment with MHCC-97H- and MHCC-97 L-derived exosomes increased the levels of phosphorylated Met, Akt and VEGFR2 compared to those of the treatment with sorafenib alone or sorafenib plus LO2-exosome groups (Fig. 8b). However, the increase in phosphorylated c-Met and Akt induced by MHCC-97H- and MHCC-97 L-derived exosomes was weakened by the c-Met inhibitor crizotinib (Fig. 8c), and the increase in phosphorylated Akt induced by MHCC-97H- and MHCC-97 L-derived exosomes was also reduced by the p-Akt inhibitor MK-2206 (Fig. 8d).

## Discussion

The tumor microenvironment plays an important role in drug resistance and tumor recurrence [24, 25], which is a major obstacle to treating advanced tumors. As a transport carrier of various biological molecules, exosomes and exosomal active factors (proteins, mRNAs, miRNAs and others) participate in the regulation of the tumor microenvironment [14, 26]. Thus, the role of exosomes as biological delivery vehicles in drug resistance is of considerable interest [27]. Previous studies suggested that a combination of guggulsterone and bexarotene reduced cellular levels of breast cancer resistance protein to 20 % of that of the control cells by inducing its association and secretion with exosomes [28]. Shao et al. [29] analyzed MGMT and APNG mRNA levels in enriched tumor exosomes obtained from blood, and they found that exosomal mRNA levels of these enzymes correlated well with levels found in parental cells and that the levels changed considerably during treatment of seven glioblastoma multiforme patients. These markers may be used to predict drug response in glioblastoma multiforme patients [29]. Moreover, drug-resistant breast cancer cells may spread resistance to sensitive cells by releasing exosomes containing specific miRNAs (miR-4443, miR-574-3p, and others) [30].

Exosomes derived from the serum of prostate cancer patients influenced cellular proliferation, invasion and response to docetaxel, which may be partly due to exosomal MDR-1/Pgp transfer [18]. Bone marrow stromal cells and multiple myeloma cells could mutually exchange exosomes carrying specific cytokines, which increased multiple myeloma cell growth and induced drug resistance to bortezomib [31]. In addition, exosomal lncRNA has recently attracted more attention. Takahashi et al. [32] found that specific exosomal lncRNA mediators, such as lincRNA-ROR, are involved in modulation of hepatoma cellular responses to sorafenib. Moreover, lincRNA-VLDLR could be transferred by HCC cell-derived exosomes and modulate resistance to anti-cancer agents, such as sorafenib, camptothecin, and doxorubicin, in recipient cancer cells [33]. Here, we showed for the first time that different invasive HCC cell-derived exosomes can regulate the sensitivity of HCC to sorafenib, in part by reversing sorafenib-induced apoptosis (Figs. 5, 6 and 7). Moreover, elevated expression of a cytokine (HGF) may be an important mechanisms underlying HCC resistance to sorafenib (Fig. 8). HCC cell-derived exosomes transfer of the soluble factor HGF may contribute to the regulation of the tumor microenvironment [34].

The HGF receptor, c-Met, is a proto-oncogene [5, 35]. Studies have found that its overexpression in tumor cells is an important mechanism of sorafenib resistance, and the c-Met-targeting drug PHA665752 inhibits its expression in various HCC cell lines. In vivo experiments also confirmed that inhibition of c-Met in HCC cells could increase their sensitivity to therapeutic agents [36]. Our previous study found that hepatic stellate cells and their conditioned medium promoted cell proliferation and enhanced the sorafenib resistance of liver cancer cells [37]. In this study, we found that treatment of HCC cells with MHCC-97 L and MHCC-97H cell-derived exosomes increased HGF levels in the medium, which further activated p-Met in liver cancer cells, ultimately leading to activation of its key downstream protein, p-Akt (Fig. 8a, b). The effect induced by HCC cell-derived exosomes was reversed by the c-Met inhibitor crizotinib and the p-Akt inhibitor MK-2206 in recipient cells. Both inhibitors indirectly or directly reduced the phosphorylation of Akt (Fig. 8c, d). Our studies confirmed that HCC cell-derived exosomes induced sorafenib resistance by increasing HGF levels in the tumor microenvironment and activating the c-Met/Akt pathway in vitro, suggesting that HGF/c-Met may be an important target for improving sorafenib resistance of HCC. Our study further revealed that HCC cell-derived exosomes promoted sorafenib resistance in liver cancer, and exosomes derived from highly invasive tumors could trigger stronger drug resistance. Mian et al. characterized the exosomal RNA and proteome contents derived from three HCC cell lines (HKCI-C3, HKCI-8 and MHCC-97 L) using Ion Torrent sequencing and mass spectrometry. RNA deep sequencing and proteomic analysis revealed that exosomes derived from different metastatic HCC cell lines had different levels of proteins and pro-tumorigenic RNAs. Thus, we hypothesized that exosomes from different sources contained different levels of cytokines (HGF, TGFβ, and VEGF) and proteins [34], which could be transported to recipient cells and played various roles in the regulation of sorafenib resistance in HCC. Moreover, differential miRNA expression in exosomes derived from different cell lines should be determined using miRNA microarrays, and the roles of these miRNAs in sorafenib resistance of liver cancer should be explored further.

## Conclusions

In summary, this study confirmed that HCC cell-derived exosomes can enhance sorafenib resistance in liver cancer cells in vitro, and exosomes derived from highly invasive tumors have greater effects than those derived from less invasive tumors. Moreover, HCC cell-derived exosomes exerted their functions by increasing the level of proteins related to sorafenib resistance, protecting tumor cells from sorafenib-induced apoptosis and activating the HGF/c-Met/Akt pathway in vitro. Our results suggest that HCC cell-derived exosomes are important mediators of sorafenib resistance in liver cancer cells. Targeting HCC cell-derived exosomes or the HGF/c-Met/Akt pathway may help improve treatment efficacy in liver cancer.
